# Verteporfin inhibits the cGAS-STING pathway and improves the tumor microenvironment during cisplatin treatment in hepatocellular carcinoma

**DOI:** 10.3389/fimmu.2025.1658042

**Published:** 2025-08-22

**Authors:** Yi Gong, Yajun Xiong, Yuting Gao, Xiaoyong Song, Yanli Gong, Dan Wang, Zhihan Liu, Yanguang Yang, Junlan Lu, Isabelle Xinyue Zou, Xinli Shi

**Affiliations:** ^1^ Laboratory of Integrated Medicine Tumor Immunology, Shanxi University of Chinese Medicine, Taiyuan, China; ^2^ Department of Pathobiology and Immunology, Hebei University of Chinese Medicine, Shijiazhuang, China; ^3^ Department of Acute General Medicine, John Radcliffe Hospital, Oxford, United Kingdom

**Keywords:** cisplatin, Yap1, hepatocellular carcinoma, PD-1, CGAS, STING, CD8 + T cells

## Abstract

**Background:**

Cisplatin (DDP) is a clinical first-line chemotherapy drug for hepatocellular carcinoma (HCC), but treatment is often ineffective due to drug resistance. Yes-associated protein 1 (YAP1) is a critical regulator/factor in HCC tumor progression. Our previous research showed that DDP promoted the expression of YAP1 in mice bearing H22 cell in situ liver tumors, which might be related to the poor therapeutic effect of DDP.

**Methods and results:**

DDP could inhibit tumor growth and decrease tumor volume in DEN/TCPOBOP-induced HCC mice, increase the number of CD8^+^ T cells in the tumor, reduce the proportion of PD-1^+^CD8^+^ T cells in the peripheral blood and spleen of mice, and reduce the immune exhaustion of the tumor microenvironment in HCC. Of note, that DDP treatment activated YAP1 expression in HCC cells. In addition, using a murine model of subcutaneous transplantation of HCC cells, it was found that the combined use of the YAP1 inhibitor, verteporfin, and DDP led to significant tumor regression. Inhibition of YAP1 reduced activation of the cGAS-STING pathway by DDP treatment. Furthermore, bioinformatics analysis revealed that YAP1 was positively correlated with cGAS and STING in HCC tissues. We further confirmed the correlation of YAP1 with cGAS-STING in HCC using two models: DEN/TCPOBOP induction of HCC in hepatocyte-specific Y*ap1* knockout mice; and giving verteporfin treatment to mice with subcutaneously transplanted HCC tumors. Inhibiting the expression of YAP1 in HCC tissues can reduce the expression of cGAS-STING and enhance the therapeutic effect of cisplatin.

**Conclusions:**

The combination of YAP1 inhibitor, verteporfin and DDP enhances anti-tumor immunity by regulating the interaction between YAP1 and cGAS-STING in the tumor microenvironment, providing new insights into a combined chemotherapy strategy for HCC.

## 1 Introduction

As is well documented, hepatocellular carcinoma (HCC) ranks as the third most common cause of cancer-related deaths worldwide (1). Surgical resection and chemotherapy remain gold standards for HCC treatment. The chemotherapeutic drug cisplatin (DDP) has been extensively administered to treat neoplasms (2). However, its clinical efficacy is often limited by the tumor’s immunosuppressive microenvironment and the development of resistance mechanisms (3). Over the years, significant efforts have been made to explore strategies to improve the therapeutic response to DDP and to overcome its limitations, such as targeting the tumor microenvironment and modulating immune checkpoint pathways (4).

The PD-1/PD-L1 pathway has been recognized as a crucial component associated with cancer immunity (5). PD-L1, an immune checkpoint molecule, is abundantly expressed in tumor cells (6, 7). PD-L1, when overexpressed, binds to PD-1 on T cells and other immune cells, thereby facilitating immune evasion (8, 9). However, the impact of cisplatin on PD-1 and PD-L1 expression remains underexplored in HCC cells.

Yes-associated protein 1 (YAP1) acts as the Hippo signaling pathway downstream component (10). There exists a close correlation between aberrant hepatocyte proliferation and the abnormal activation of YAP (11). Furthermore, studies have linked YAP1 to key immune-related pathways, including cGAS-STING, a crucial pathway involved in the innate immune response (12, 13). However, the precise role of YAP1 in regulating immune responses during chemotherapy and its potential as a therapeutic target in HCC remains poorly understood.

The cyclic GMP-AMP synthase-stimulator of interferon genes (cGAS-STING) axis, an essential signaling pathway in innate immunity, is a DNA-sensing pathway that has been central to our understanding of intrinsic anti-tumour immunology in recent years. Cisplatin belongs to a class of chemotherapy drugs that can cause DNA breakage and damage in tumor cells. Therefore, cisplatin may induce tumor cells to activate endogenous cGAS-STING, which plays an anti-tumor role (14).

Overall, this study highlights the interaction of YAP1 with a key immune pathway, cGAS-STING. Our findings suggest that inhibiting YAP1, in combination with DDP, can modulate immune responses and enhance the therapeutic efficacy of chemotherapy in HCC. These results offer new insights into potential combination therapies for improving the clinical outcomes of HCC treatment.

## 2 Materials and methods

### 2.1 Animal

All animals were maintained under SPF conditions at a constant temperature (22-24°C) and housed in plastic cages. Hepatocellular-specific *Yap1* knockout mice were bred and confirmed by Guangzhou Cyagen Biosciences (Guangzhou, China). Hepatocellular-specific *Yap1* knockout mice were denoted as *Yap1* ^LKO^ (with the genotype *Yap1* ^Flox/Flox^; albumin-cre) and *Yap1* ^Flox^ mice (with genotype *Yap1* ^Flox/Flox^).

The DEN/TCPOBOP modeling method was implemented following the methodology outlined in the study by Bergmann et al. (15). Mice were weighed, numbered, and randomly divided into four groups of 5 mice each. Following the successful establishment of the model, Mice in the DDP group were administered DDP dissolved in saline, whereas control mice were injected with saline alone.

The establishment method of subcutaneous tumor transplantation was referred to in the previous article (16). 5×10^6 Hepa1-6 cells were injected into the subcutaneous skin of the right hind limb of 4-6 weeks C57BL/6N mice. The dose of verteporfin was 100mg/kg, once every three days, and the dose of DDP was 2mg/kg, once every two days and the intervention time was 14 days. All experimental protocols involving animals were approved by the Ethics Committee for Animal Experimentation at Hebei University of Chinese Medicine (Permit number: DWLL202203064).

### 2.2 Flow cytometry

Mouse spleen tissue was placed in lymphocyte separation medium, then crushed with a grinding rod and filtered to obtain a spleen cell suspension. Mouse peripheral blood cells were processed by adding whole blood into an anticoagulant tube with red blood cell lysis buffer and incubating at room temperature in the dark for 20 minutes, to remove red blood cells. To 100μl of the processed lymphocyte samples, 1 μl of FITC Anti-Mouse CD3 antibody (553061, BD), 5 μl of PerCP-Cy5.5 Anti-Mouse CD8 antibody (551162, BD), and 5 μl of PE Anti-Mouse PD-1 antibody (551892, BD) were added sequentially. The samples were stained at 4°C for 20 minutes.

Using the blank tubes and single-stained tubes, the voltage was set and threshold and compensation were adjusted. According to the forward scatter (FSC) and side scatter (SSC) characteristics of the cells, a rough gating was first performed on the cells. FSC-A vs SSC-A was used to exclude debris and select intact lymphocytes. FSC-H vs FSC-A was used to exclude doublets and select single cells. For specific immune cell populations, we used the corresponding surface markers for precise gating. CD3 positivity allowed for selection of T cells. CD8 and PD-1 positivity allowed the PD-1+CD8+ cell subset to be analyzed.

### 2.3 Hematoxylin and eosin staining

Upon organ collection, 4% formaldehyde was applied for 48 hours to the liver and subcutaneous graft tumor tissues before being embedded in paraffin and sectioned at a thickness of 4 µm. The sliced sections were subsequently stained with H&E, and histopathological alterations were visualized under a microscope (Leica DM2255, Germany) at 100×/200× magnification.

### 2.4 Western blot

Extraction of mice liver protein, BCA method for the determination of protein concentration. Sample addition, electrophoresis. Thereafter, the isolated proteins were transferred to PVDF membrane, and then 5% skim milk was added for four hours at ambient temperature. Following this, the membrane was incubated with the primary antibodies: YAP1 antibody (1:1000, CST,14074s),.cGAS antibody (1:1000, Proteintech, 29958-1-AP), STING antibody (1:1000, CST, 13647s), GSDMD antibody (1:1000, Proteintech, 20770-1-AP) and GAPDH antibody (1:5000, Abways, AB0037).

Then, a secondary antibody was incubated with the membrane, namely goat anti-rabbit IgG-HRP (1:5000, Absin, abs20002). ECL luminescent solution was added to the strip (share-bio, SB-WB012).

### 2.5 Immunohistochemistry

Immunohistochemistry (IHC) staining was conducted following the steps outlined in the Nanjing Zhongshan Jinqiao immunohistochemical kit. Ki67 antibody (1:150,CST, #34330). The remaining primary antibodies were the same as those used for Western blotting. Finally, two senior histopathologists analyzed the sections under a microscope at 200× and 400× magnification.

### 2.6 Bioinformatics analysis

We used the TIMER and GEPIA tools to analyze the association between YAP1, cGAS (C6ORF150/MB21D1), and STING(TMEM173) in HCC patients in the TCGA database.

### 2.7 Statistical analysis

Statistical analyses were conducted using SPSS23.0. Data were presented as Mean ± SD. Two groups were compared using a student’s t-test. When more than two groups were included, one-way ANOVA was used. *P*<0.05 was identified as statistically significant.

## 3 Results

### 3.1 DDP reduced PD-1^+^CD8^+^ T cell numbers in the liver tumor niche and up-regulated hepatic YAP1 expression

**Figure 1:**
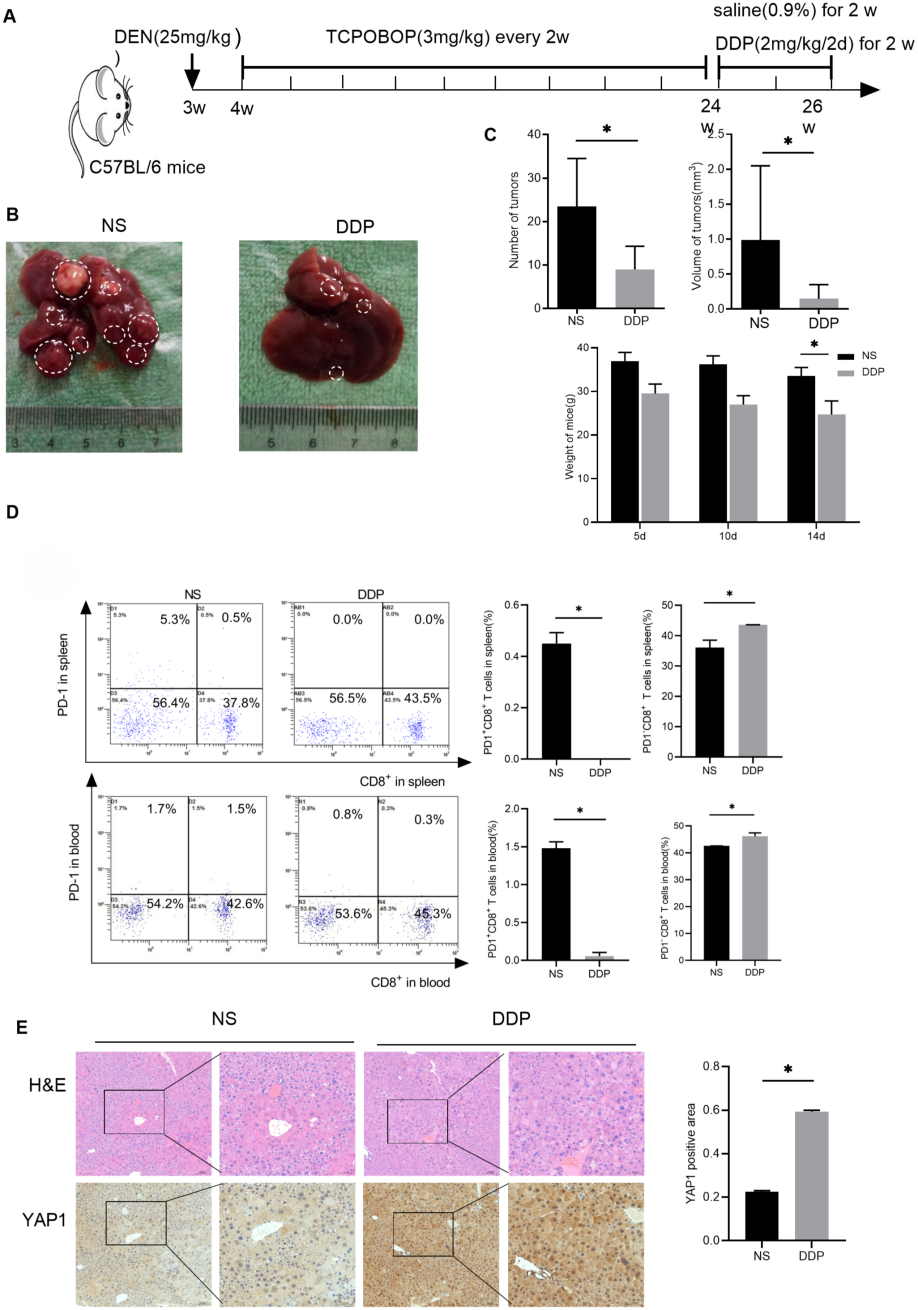
DDP reduced PD-1^+^CD8^+^ T cell numbers in the liver tumor niche and up-regulated hepatic YAP1 expression. **(A)** Flow chart of modeling DEN/TCPOBOP-induced HCC mice. DEN/TCPOBOP-induced HCC mice were given DDP and saline treatment. **(B)** Images of DEN/TCPOBOP-induced HCC mouse liver. Tumors are represented by white circles. **(C)** Tumor number and volume, as well as mouse weight, were measured. **(D)** Flow cytometry detected the number of CD8^+^ and PD-1^+^ cells in the spleen and blood of mice, the number of PD-1^+^CD8^+^T cells and PD-1^-^CD8^+^T cells were counted in the histogram. **(E)** Detection of YAP1 expression in the liver of DEN/TCPOBOP-induced HCC mice by IHC and the expression was statistically analyzed by bar chart. Data are presented as mean ± SD, *P<0.05.

Our previous research established that DDP promoted YAP1 expression in the liver of BALB/c mice loaded with H22 cells (17). To evaluate the effect of DDP, an *in situ* liver tumor mouse model was established following the method described above (**Figure 1A**). Interestingly, the saline group (NS) of mice liver visible large tumors. In contrast, no apparent tumors were observed in the liver of mice in the DDP group (**Figure 1B**). While DDP reduced the number of tumors, it also decrease the tumor volumes and weight of mice (**Figure 1C**). In conclusion, DDP inhibits liver tumor growth in C57BL/6 mice.

In anti-cancer responses, CD8**^+^** T cells play an instrumental role (18, 19). Our previous research indicates that that DDP fostered CD8^+^ T cell infiltration in the liver cancer microenvironment (20). Additionally, flow cytometry suggested that DDP reduced exhausted CD8^+^T lymphocytes (PD-1^+^CD8^+^ T cells) proportion and enhanced that of PD-1^-^CD8^+^ T cells in the spleen and blood (**Figure 1D**). These results conjointly suggested that DDP-promoted CD8^+^ T cell infiltration and decreased exhausted CD8^+^ T lymphocytes in the blood and spleen. Finally, immunohistochemistry analysis determined that DDP treatment up-regulated YAP1 expression in HCC mice (**Figure 1E**).

### 3.2 Verteporfin,YAP1 inhibitor, is more effective for HCC treatment

**Figure 2:**
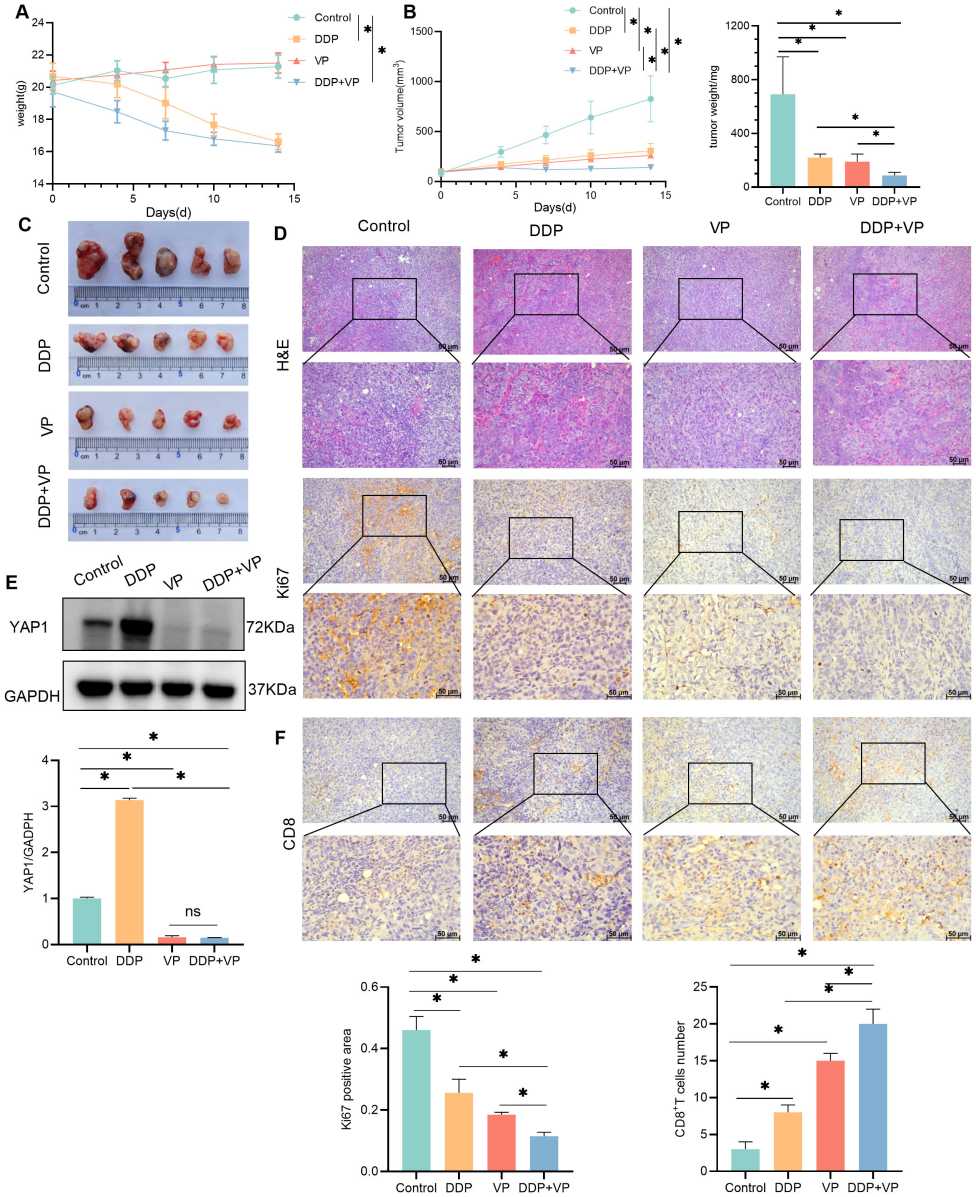
Verteporfin,YAP1 inhibitor, is more effective for HCC treatment. **(A)** Changes of body weight in control, DDP, VP, DDP +VP groups of mice. **(B)** Changes of tumor volume and final tumor weight in control, DDP, VP, DDP +VP groups of mice. **(C)** Photograph of tumors excised from subcutaneous transplanted tumor mice. **(D)** H&E and IHC detection of Ki67 expression in transplanted tumors. The expression was statistically analyzed in the histogram. **(E)** Western blot detection of YAP1 expression in transplanted tumors, as well as GAPDH as a loading control. The expression was statistically analyzed by bar chart. **(F)** IHC detection of CD8 expression in transplanted tumors, CD8^+^T cells were counted in the histogram. Data are presented as mean ± SD, *P<0.05.

In order to analyze the mechanism of DDP treatment for liver cancer more comprehensively, we also established another liver cancer model-Hepa1-6 hepatocellular carcinoma bearing mouse model. Randomly divided into four group -control group, DDP group, YAP1 inhibitor -Verteporfin (VP) group, and DDP combined with Verteporfin group. We found that both DDP and Verteporfin, as well as the combination of DDP and Verteporfin effectively inhibited subcutaneous transplanted tumors of mouse hepatocellular carcinoma cells growth. After drug intervention, H&E staining showed that the nucleus of the tumor tissue became sparse and no longer so dense, and the Ki67 expression of immunohistochemistry showed that the proliferation ability of the tumor mass became weak after drug intervention, in which the combination of DDP and Verteporfin had the better therapeutic effect (**Figures 2A–D**). Western blot results showed that DDP could promote the YAP1 expression in subcutaneously transplanted tumors, but under the premise of using Verteporfin, DDP could not promote YAP1 expression in subcutaneously transplanted tumors. (**Figure 2E**). The IHC results indicated that both DDP and Verteporfin used alone could increase CD8^+^T cell numbers in the tumor mass tissue, and CD8^+^T cell numbers in the tumor mass tissue increased even more after combined use (**Figure 2F**).

### 3.3 Combining verteporfin with DDP reduced cGAS-STING expression

**Figure 3:**
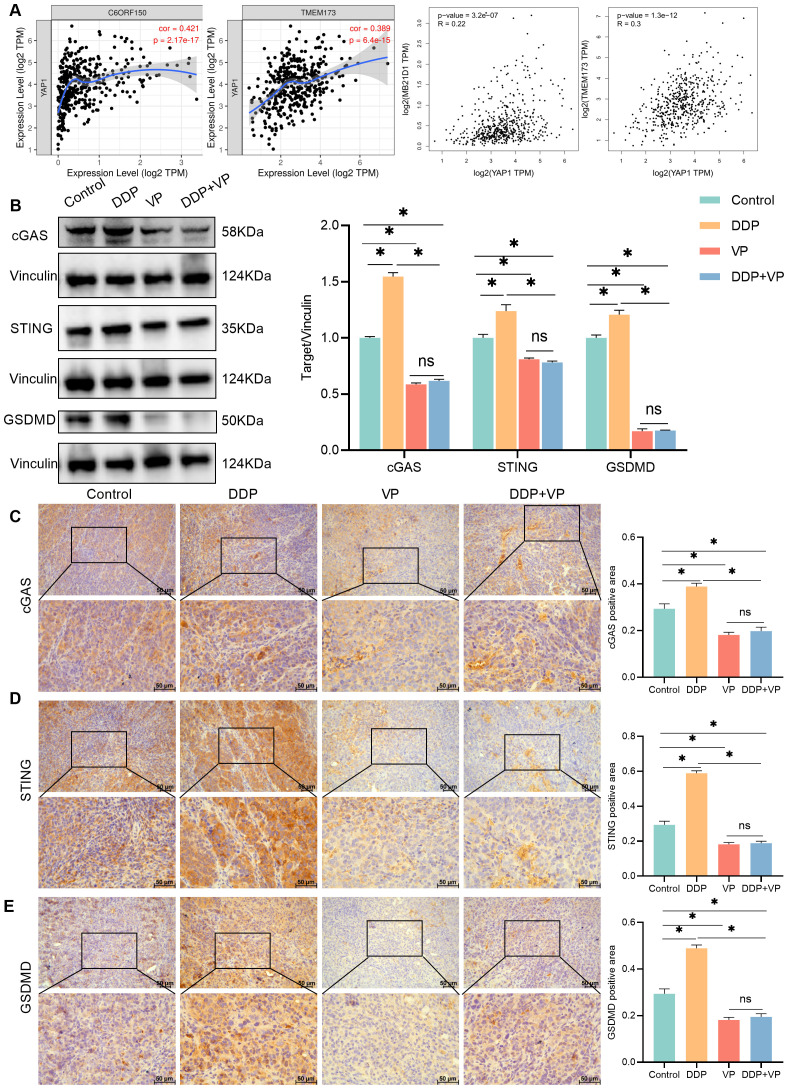
Combining verteporfin with DDP reduced cGAS-STING expression. **(A)** The correlation between YAP1 and cGAS(C6ORF150/MB21D1), STING(TMEM173) were analyzed by TIMER/GEPIA. **(B)** Western blot detection of cGAS,STING and GSDMD expression in transplanted tumors, as well as Vinculin as a loading control. **(C–E)** IHC staining for cGAS/STING/GSDMD expression in transplanted tumors. Data are presented as mean ± SD, *P<0.05.

The relationship between cGAS-STING and liver cancer was introduced in a study by Li et al (14). Through bioinformatics analysis, we found that YAP1 was positively related to cGAS and STING in liver cancer (**Figure 3A**) (cor/R>0 and *P*<0.05). Western blot analysis showed that compared with the control group, DDP increased the abundance of cGAS and STING proteins. The abundance of cGAS and STING proteins was decreased by both verteporfin and the combination of DDP and verteporfin (**Figure 3B**).

Increased activation of the cGAS-STING pathway in cancer cells limits tumorigenesis, through the upregulation of inflammatory genes, which leads to the release of inflammatory cytokines and chemokines that slow down tumor growth and recruit anti-tumor immune cells (21). Gasdermin D (GSDMD) is a pyrogenic enforcer and GSDMD-induced pyrogenic cell death enhances the immune defense function (22). Compared to the control group, DDP increased GSDMD expression. Verteporfin inhibited GSDMD expression. The combination of Verteporfin and DDP had no impact on GSDMD expression (**Figures 3B, E**).

Our findings also confirmed that YAP1 was positively related to cGAS, STING, and GSDMD in liver cancer tissues. The results from IHC analysis were consistent with Western blot (**Figures 3C–E**). We therefore concluded that DDP stimulated cGAS/STING/GSDMD expression, whereas verteporfin inhibited the Hippo-YAP signaling pathway. The combined action of inhibiting the cGAS/STING pathway and YAP1 results in a better therapeutic effect on liver cancer. The decrease in expression of GSDMD following treatment with verteporfin and DDP together was not statistically different to that for verteporfin alone (**Figures 3B, E**).

### 3.4 YAP1 hepatocellular-specific knockout reduced the tumor size during DDP treatment

**Figure 4:**
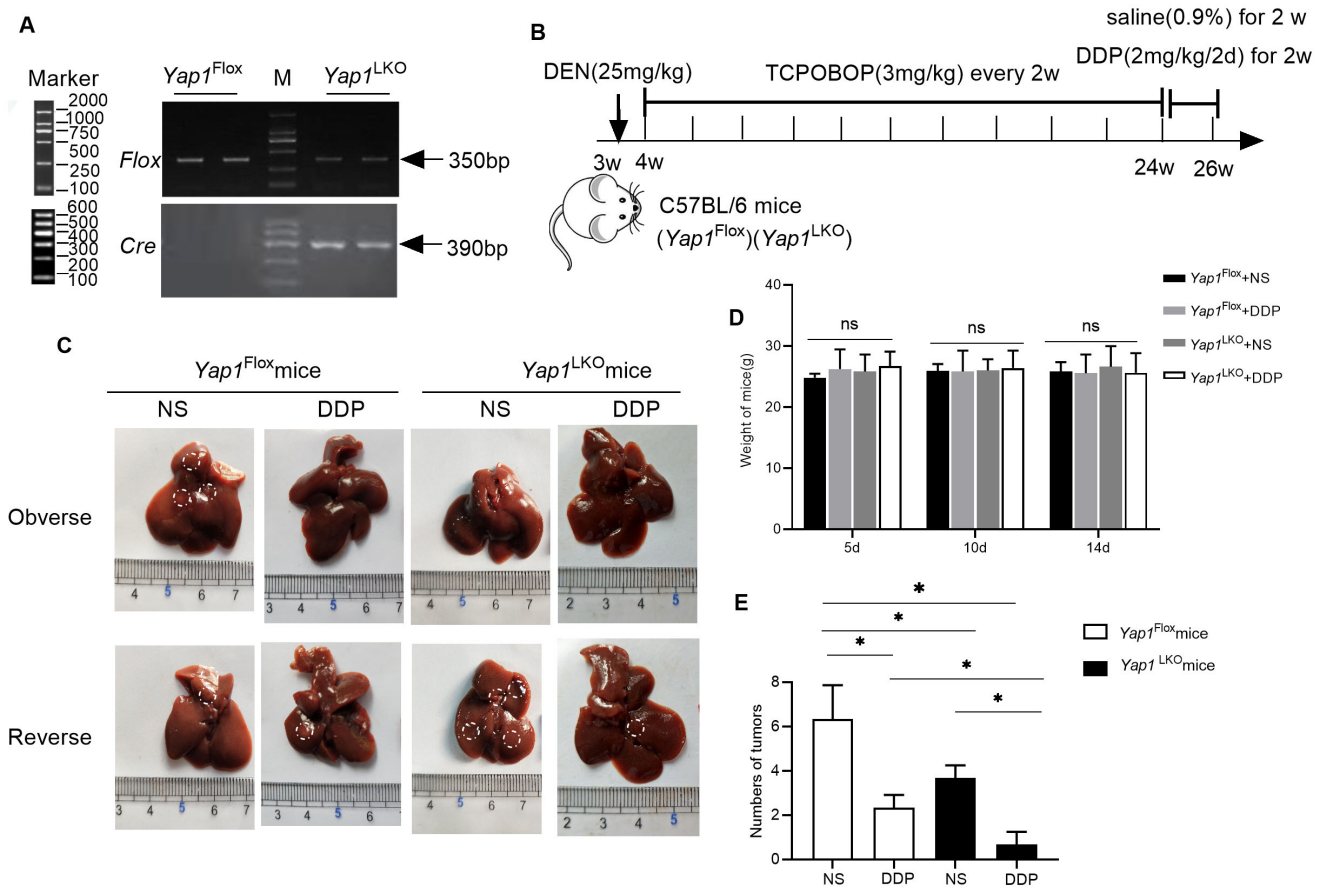
YAP1 hepatocellular specific knockout reduced the tumor size during DDP treatment **(A)** The expression of *Flox* and *Cre* was detected by PCR. **(B)** Schematic representation of the mouse model. **(C)** Macroscopic view of the liver specimens with visible tumors highlighted. (“Observe” and “Reserve“ represent the front and back sides of the gross liver image). **(D)** Fluctuations in body weight during the intervention. **(E)** The number of tumors on the liver after sampling. Data are presented as mean ± SD, *P<0.05.

To investigate the mechanism by which DDP alters PD-L1 and PD-1 expression, and how YAP1 is associated with this, experiments were conducted in hepatocellular specific *Yap1* knockout (*Yap1* ^LKO^) and control (*Yap1* ^Flox^) C57BL/6 mice. PCR detected *Flox* and *Cre* expression to identify the genotype of mice (**Figure 4A**). *Yap1* ^LKO^ and *Yap1* ^Flox^ mouse models were established following the method described above (**Figure 4B**). As anticipated, DDP treatment yielded a reduction in the number of tumors in both the *Yap1* ^Flox^ and *Yap1* ^LKO^ groups. Following treatment with DDP, treatment effect was greater in *Yap1* knockout mice, compared to in the control group (**Figures 4C–E**).

### 3.5 DDP increased PD-L1 expression and decreased PD-1 expression *via* YAP1

**Figure 5:**
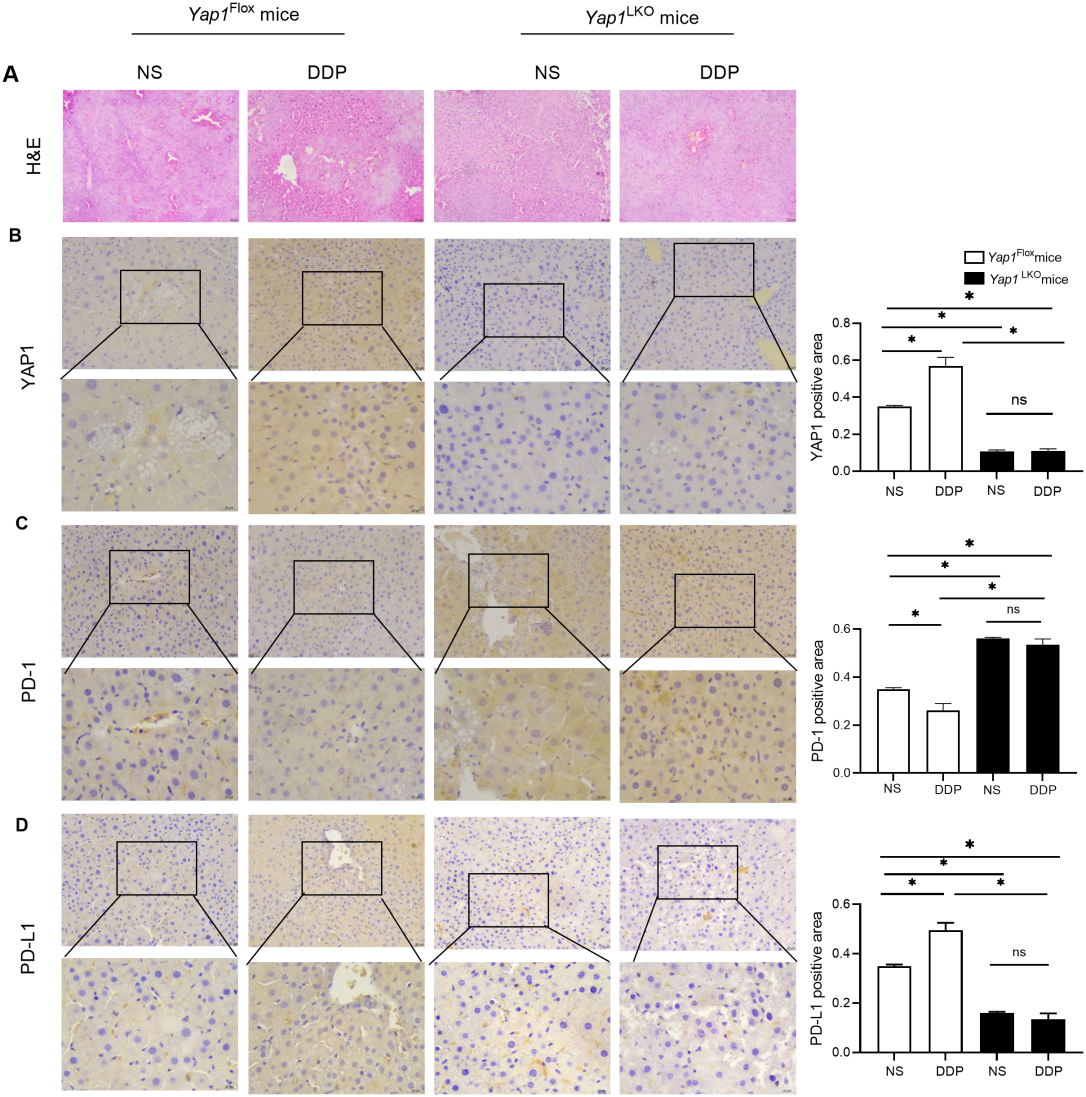
DDP increased PD-L1 expression and decreased PD-1 expression *via* YAP1. **(A)** H&E in liver of HCC mice. **(B)** YAP1 expression in liver of HCC mice was detected by IHC. **(C)** PD-1 expression in liver of HCC mice was detected by IHC. **(D)** PD-L1 expression in liver of HCC mice was detected by IHC. Data are presented as mean ± SD, *P<0.05.

Furthermore, DDP treatment increased YAP1 and PD-L1 expression while decreasing that of PD-1 in *Yap1* ^Flox^ mice. On the other hand, when *Yap1* was knocked out in liver cancer tissues, PD-L1 was decreased and PD-1 expression was increased. In hepatocellular-specific *Yap1* knockout mice, DDP no longer increased PD-L1 expression or reduced PD-1 expression in liver cancer. These results demonstrated that DDP increased PD-L1 expression and decreased PD-1 expression via the action of YAP1 (**Figures 5A–D**).

### 3.6 DDP activated cGAS-STING by YAP1

**Figure 6:**
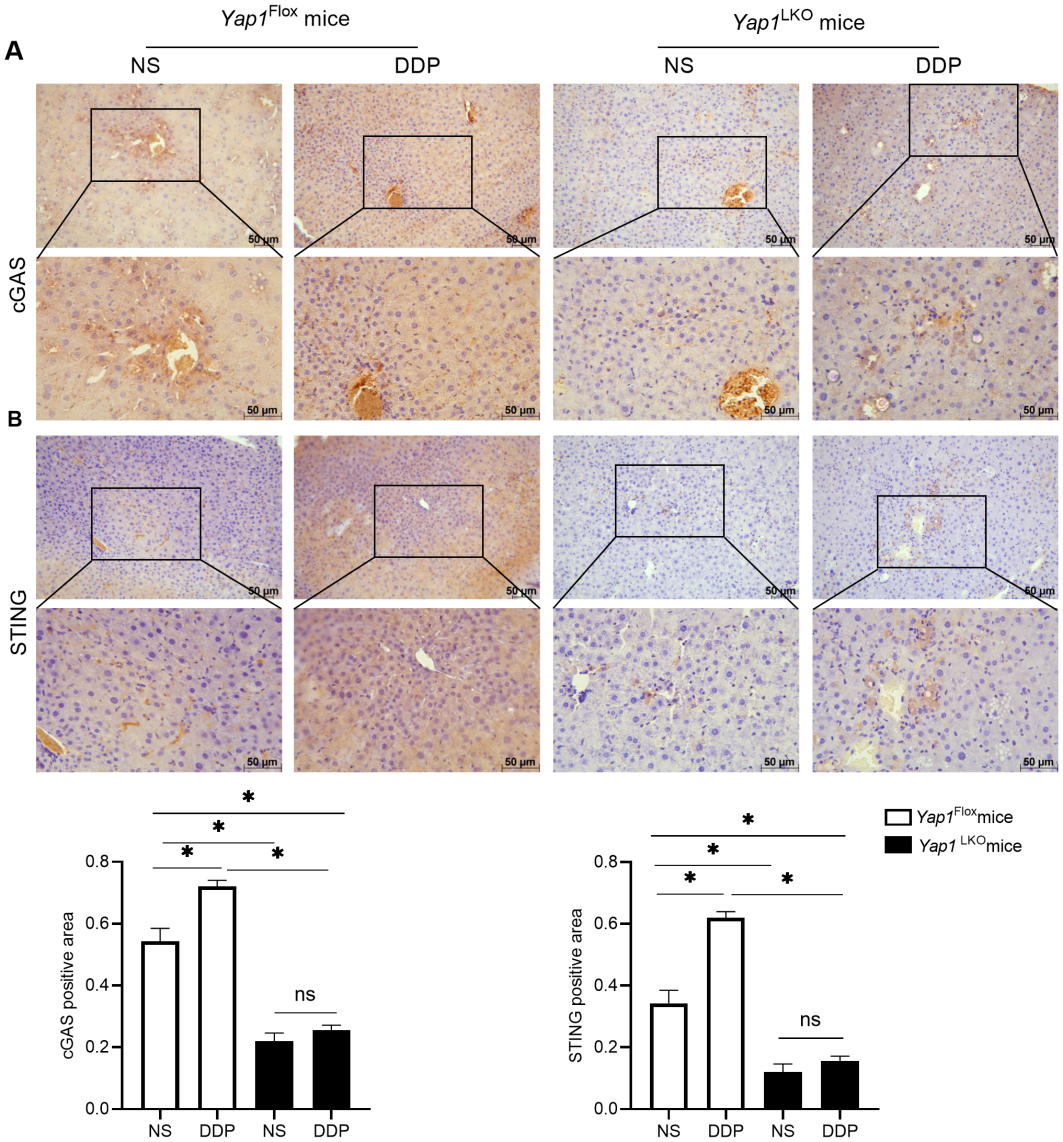
DDP activated cGAS-STING via YAP1. **(A)** cGAS expression was detected by IHC in liver of HCC mice. **(B)** STING expression was detected by IHC in liver of HCC mice. Data are presented as mean ± SD, *P<0.05.

To verify the correlation between YAP1 and cGAS-STING, we used hepatocellular-specific *Yap1* gene knockout mice to establish liver *in situ* tumour-bearing mice using DEN/TCPOBOP induction. The expression of cGAS and STING was detected using immunohistochemistry. DDP increased the expression of cGAS and STING in liver cancer tissue. When *Yap1* was knocked out in liver cancer tissues, the expressions of cGAS and STING decreased. With *Yap1* expression knocked out, DDP no longer increased the expressions of cGAS and STING in liver cancer. These results demonstrated that DDP activated cGAS-STING expression by through the action of YAP1 (**Figures 6A, B**).

## 4 Discussion

The pan-cancer analysis found that activation of YAP, a key downstream molecule of the Hippo pathway, was positively correlated with tumor-infiltrating cell abundance in several cancer types (23, 24). Previous research has indicated correlation between the expression levels of YAP1 and the infiltration of CD8^+^ T cells into liver cancer tissues (16). Furthermore, YAP1 may indirectly influence the migration, activation, and cytotoxicity of CD8^+^ T cells by modulating the expression of other immune-related molecules, such as cytokines and chemokines (16, 25).

PD-1 is universally considered a key T-cell exhaustion marker. Consequently, an increase in PD-1 expression eventually leads to CD8^+^ T cell dysfunction (26). Previous studies have demonstrated that the expression of PD-L1 in HCC is significantly associated with immune evasion, impairing the immune response against HCC. We found that PD-1 expression was reduced and PD-L1 expression was increased when YAP1 was knocked out *in vivo*. According to a previous study, YAP1 specifically binds to the PD-L1 primer, promoting PD-L1 transcription (27).

**Figure 7:**
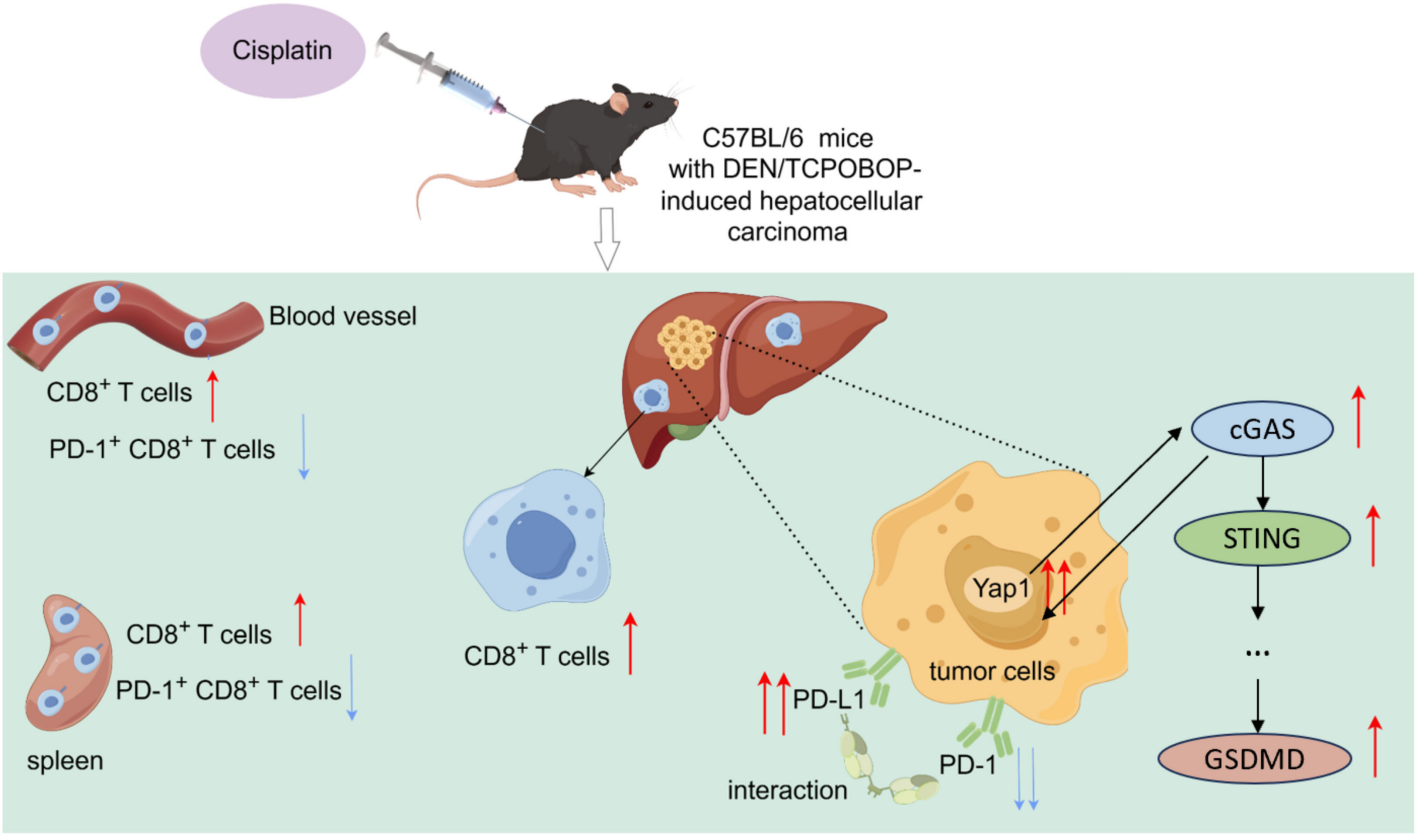
YAP1, cGAS and STING expression were increased in the liver of DEN/TCPOBOP-induced HCC mice after cisplatin administration. Furthermore, cisplatin treatment resulted in an increased number of CD8+T cells in both the spleen and peripheral blood of the mice while decreasing the number of PD-1^+^CD8^+^T cells. Moreover, there was an increase in the number of CD8^+^T cells in the liver. DDP increased the PD-L1 expression through YAP1 and downregulated that of PD-1.

Cisplatin has been widely used for the treatment of cancers. Indeed, it is globally administered as a chemotherapeutic agent to patients with advanced HCC (28, 29). One of the core findings of this study is that DDP treatment significantly activates the expression of YAP1 in DEN/TCPOBOP-induced HCC mice and leads to a reduction in the number of PD-1^+^CD8^+^ T cells in both the spleen and peripheral blood of mice. Moreover, CD8^+^T cells increased in HCC (**Figure 7**). This indicates that DDP may play a dual role in regulating tumor growth and the immune response in the tumor microenvironment. This finding was in line with a prior study that documented that DDP promoted CD8^+^ T cell infiltration in E.G7-OVA tumor tissues (30). It is worwhile emphasizing that higher abundance of PD-1^+^CD8^+^T cells are associated with adverse outcomes in multiple cancers including HCC (31–35).

The cGAS/STING signaling pathway connects innate and adaptive immunity, so its overreactive state has been characterized as a facilitating factor in various tumors (13). Multiple studies have shown that directly stimulating the cGAS-STING axis in the tumor microenvironment results in significant tumor regression and more effective immune responses across a variety of tumor models, suggesting that the cGAS-STING pathway holds promise as a healing target for liver cancer treatment (36). YAP1 plays an important role in modulating immune responses by influencing the cGAS-STING pathway and expression of immune checkpoint molecules in the liver tumor microenvironment (13). And other studies have shown that YAP1 and cGAS-STING are positively correlated in non-small cell lung cancer (13). YAP1-mediated regulation may contribute to the suppression of immune activation, which, in turn, impacts the anti-tumor response to chemotherapy (37, 38). Importantly, the combination of verteporfin and DDP not only enhanced the immune response but also reduced immune exhaustion in the tumor microenvironment, indicating YAP1 as a therapeutic target to overcome immune suppression during chemotherapy.

At present, there are no clinical reports of the combined use of YAP inhibition and cisplatin in the treatment of liver cancer or other tumors. Our research provides a preliminary investigation of the combined use of YAP inhibition and cisplatin in the treatment of liver cancer. Other studies have shown that in ovarian cancer and small cell lung cancer, activation of YAP1 can lead to cisplatin resistance. As a YAP1 inhibitor, verteporfin can inhibit cisplatin resistance by suppressing YAP1, thereby enhancing the efficacy of cisplatin (39, 40).

This study only focused on the improvement of liver cancer therapeutic efficacy by the combined use of cisplatin and verteporfin. It did not establish cisplatin-resistant liver cancer cell lines or cisplatin-resistant mice. Experimental research has been conducted on the correlation between YAP1 and cisplatin resistance in liver cancer treatment, as well as the issues of nephrotoxicity and weight loss in mice during cisplatin treatment. We aim to conduct further research on these limitations of cisplatin in the future.

In conclusion, our study provides compelling evidence that targeting YAP1 in combination with DDP can significantly enhance the immune response improve the efficacy of chemotherapy in HCC and enhance CD8^+^T cells in liver cancer tissues. By regulating the interaction between YAP1 and the cGAS-STING pathway, we demonstrate that inhibition of YAP1 can overcome immune suppression and enhance anti-tumor immunity, offering new therapeutic strategies for HCC treatment. Other studies have also confirmed the interaction between YAP and cGAS-STING pathways demonstrated previously in lung cancer tissues (13), Our findings also support that of current evidence in transplanted mouse tumor models for bladder cancer, showing that cGAS-STING signaling induced by cisplatin inhibits tumor proliferation and boosts CD8^+^ T cell infiltration (41). Additionally, activation of STING inhibits tumor-derived antigens from being incorporated into CD8^+^ T cells during *in vivo* immune responses (36, 42).

## Data Availability

The original contributions presented in the study are included in the article/supplementary material. Further inquiries can be directed to the corresponding author.
